# Micro-Fabricated Presure Sensor Using 50 nm-Thick of Pd-Based Metallic Glass Freestanding Membrane

**DOI:** 10.1038/s41598-020-67150-y

**Published:** 2020-06-22

**Authors:** Nguyen Van Toan, Truong Thi Kim Tuoi, Yao-Chuan Tsai, Yu-Ching Lin, Takahito Ono

**Affiliations:** 1 Microsystem Integration Center (μSIC), Tohoku University, Sendai, Japan; 2 Department of Mechanical Systems Engineering, Graduate School of Engineering, Tohoku University, Sendai, Japan; 3 Department of Bio-industrial Mechatronics Engineering, National Chung Hsing University, Taichung, Taiwan; 4 Goertek Technology Japan Co., Ltd., Tokyo, Japan

**Keywords:** Materials science, Nanoscience and technology

## Abstract

This paper reports on micro-fabricated pressure sensors based on a thin metallic glass membrane. The Pd_66_Cu_4_Si_30_ metallic glass material is deposited successfully by a sputter technique. An amorphous feature of the deposited film is confirmed by high resolution transmission electron microscopy (HR-TEM) and the corresponding the selected area electron diffraction (SAED). The ultra-flat freestanding metallic glass membrane with 50 nm in thickness and 2 mm in circular diameter has been fabricated successfully. In addition, two kinds of micro-fabricated pressure sensor types, including itself membrane and additional metallic glass bar as piezoresistive sensing elements, are proposed and fabricated. A displacement of membrane can reach over 100 µm without any damage to membrane which is equivalent to over 0.7% of an elastic strain. Besides, the temperature coefficient of resistance of the Pd-based metallic glass thin film is extremely low 9.6 × 10^−6^ °C^−1^. This development of nano-thick metallic glass membrane possibly opens a new field of micro-fabricated devices with large displacement and enhanced sensing.

## Introduction

Micro/nano fabricated devices play an essential role in a variety of applications.^1–6^ Especially, Micro-fabricated pressure sensors have been commonly employed in many fields, including automobile,^7–9^ aerospace^10,11^ and biomedical^12–14^ applications for pressure measurements. Micro-fabricated pressure sensors can be divided into capacitive,^15^ piezoresitive,^16^ optical,^17^ and other types.^18,19^ Among them, piezoresisitve pressure sensors are most employed because of their high performance, high sensitivity, small size, and low cost. Until now, silicon material is still being used to fabricate micro pressure sensors due to good sensitivity, high mechanical stability and mass production capability process. The main components of Piezoresistive micro-fabricated pressure sensors consist of piezoresistive sensing elements and a thin membrane. When a pressure is applied, the membrane is deformed which causes the change of the resistance of the piezoresisitve elements. The measurement range of pressure is as a function of the dimensions of the membrane. There is the trade-off between sensitivity and linearity of output, especially in a low pressure range^20,21^ which is employed for fields such as biomedical (pulse wave and respiration rate),^22^ and smart homes.^23^ In order to use for low pressure applications, the sensitivity of MEMS device must be high to maintain an appropriate sensor output for the signal processing circuits. The sensitivity is proportional to the ratio between membrane diameter and membrane thickness. Therefore, the sensitivity can be increased by a larger diameter membrane with thinner membrane thickness. Consequently, ultra-thin membranes with large diameter are required to open the new areas of applications.

With the current micro/nano fabrication technologies, there is the possibility to reduce the thickness of silicon membrane under approximately 100 nm; however, the fabrication cost will increase and fabrication process becomes complicated. In addition, single crystal silicon and polycrystalline silicon show brittle and fracture characteristics. As such these disadvantages, micro-fabricated silicon materials render them unsuitable for use as mechanical elements with the requirement of the larger displacement. One of candidate materials to replace for silicon material is metallic glass material which is amorphous material containing the characteristics of excellent mechanical properties, high strength, and large elastic strain. Thin film metallic glass material evaluations were conducted in.^24–27^ Development of applications, including hydrogen sensors,^28,29^ implantable medical devices,^30^ and micro-mirrors,^31–33^ utilizing metallic glass materials have been developed successfully.

The aim of this research is to develop nano-thick metallic glass membranes for the application of a high sensitive micro-fabricated pressure sensor in the low pressure range. Pd-based metallic glass is deposited by a sputtering method and its amorphous characteristic is confirmed by HR-TEM and SAED. Two kinds of micro-fabricated pressure devices are proposed, fabricated and evaluated.

## Device Structure

**Figure 1:**
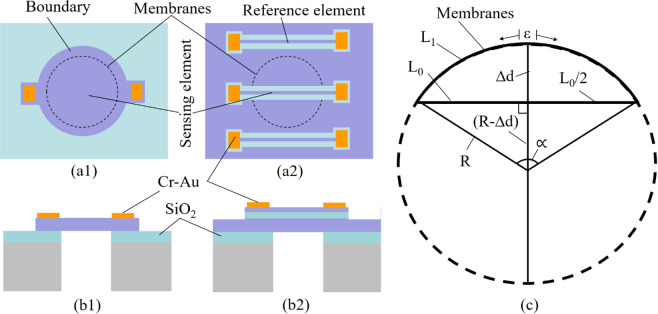
Device structure. (**a1**) Top view of Type #1 – membrane as sensing element. (**b1**) Cross sectional view of Type #1 – membrane as sensing element. (**a2**) Top view of Type #2 – thin metallic glass bar as a sensing element. (**b2**) Cross sectional view of Type #2 – thin metallic glass bar as a sensing element. (**c**) Strain estimation.

Two kinds of micro-fabricated pressure types are proposed, including type #1-membrane as sensing element and type #2-thin metallic bar on top of the metallic glass membrane as sensing element, as shown in Fig. 1 (a1,a2), respectively. For type #1, device structure consists of silicon substrate, SiO_2_ layer as insulator layer, freestanding metallic glass membrane, and Cr-Au layers as electrode (Fig. 1 (b1)) while for type #2, an additional thin SiO_2_ on top metallic glass membrane is deposited to separate sensing and reference elements with the metallic glass membrane (Fig. 1 (b2)). The sensing element as well as the reference element are the thin metallic glass bar.

Bending of a circular membrane with large displacement could be estimated,^34^ as following equation.

1 $${w}_{0}=0.662{t}^{3}\sqrt{\frac{P{r}^{4}}{E{t}^{4}}}$$

where *w*_o_ is the vertical displacement of the central point of the circular membrane. *P* is the differential pressure between two sides of the membrane. *E* is the Young module. r and *t* are radius and the thickness of the membrane, respectively.

When the metallic glass membrane deforms which caused by the applied force, the electric resistance of the sensing element will be changed. The resistance change ratio *∆R/R* could be estimated by^35^:

2 $$\frac{\Delta {\rm{R}}}{R}=\frac{\Delta \rho }{{{\rm{\rho }}}_{0}}+\frac{\Delta {\rm{l}}}{{l}_{0}}-\frac{\Delta {\rm{t}}}{{t}_{0}}-\frac{\Delta {\rm{w}}}{{w}_{0}}$$

where *ρ* is the resistivity of metallic glass material, *l* and *w* is the sensing element length and width, respectively.

Herein, the material resistivity is assumed to show no variation in membrane deformation. The change of thickness of sensing element is also eliminated. Thus, the resistance change of sensing element is contributed by the length and width of the sensing element. When the length value is much larger than width value, the width contribution could be ignored. Figure 1 (c) shows the bending situation of thin metallic glass membrane with the sensing bar on its top surface (Fig. 1 (a2)) where *L*_0_ and *L*_1_ are the length of the piezoresistive element in flat and bending conditions. *∆d* is displacement distance of the membrane. *R* and ∝ are the curvature radius and central angle, respectively. The strain (ε) in length direction of sensing element can be expressed by:

3 $$\varepsilon =\frac{{L}_{1}-{L}_{0}}{{L}_{0}}=\frac{\frac{\Pi \propto R}{180}-{L}_{0}}{{L}_{0}},$$

where ∝ and *R* could be estimated from the information of displacement *∆d* of the membrane.

The strain gauge factor (*GF*) is defined as the ratio of fractional change in an electrical resistance to the fractional change in a mechanical strain, expressed as below:

4 $$GF=\frac{\Delta R/{R}_{0}}{\varepsilon }$$

## Experimental

### Metallic glass material

The preparation of the metallic glass layer used here is described in details, as following: Silicon substrate are diced from 4 inch silicon wafer into several pieces of 2 cm by 2 cm, and cleaned by a conventional method, including Piranha solution (H_2_SO_4_:H_2_O_2_ = 2:1, to remove organic particles), RCA1 (NH_4_OH:H_2_O_2_:H_2_O = 1:1:6, to remove any possible remaining organic contamination) and RCA2 (HCl:H_2_O_2_:H_2_O = 1:1:6, to etch the inorganic contamination such as some kinds of nano metal particles) to remove any possible organic contamination and metal particles. A 200 nm-thick SiO_2_ film is deposited on this wafer by chemical vapor deposition (CVD). Next, the PdSiCu metallic glass thin film is deposited at room temperature on the SiO_2_ surface by a magnetron sputtering method. The base chamber pressure is maintained at 3 × 10^−6^ Pa and sputter pressure of Ar gas is 0.4 Pa during the deposition process. The sputtering radio frequency (RF) power is set at 100 W. The sample holder is rotated throughout the deposition process to ensure the deposited film uniformity and the film thickness is controlled at around 50 nm. The composition of the films was measured using the energy dispersive X-ray spectrometer (EDX) attached to scanning electron microscopy (SEM, Hitachi SU-70) at an accelerating voltage of 15 kV. The atomic lattice and amorphous feature of deposited film is examined using a high-resolution transmission microscope (HR-TEM) and selected area electron diffraction (SEAD).

**Figure 2:**
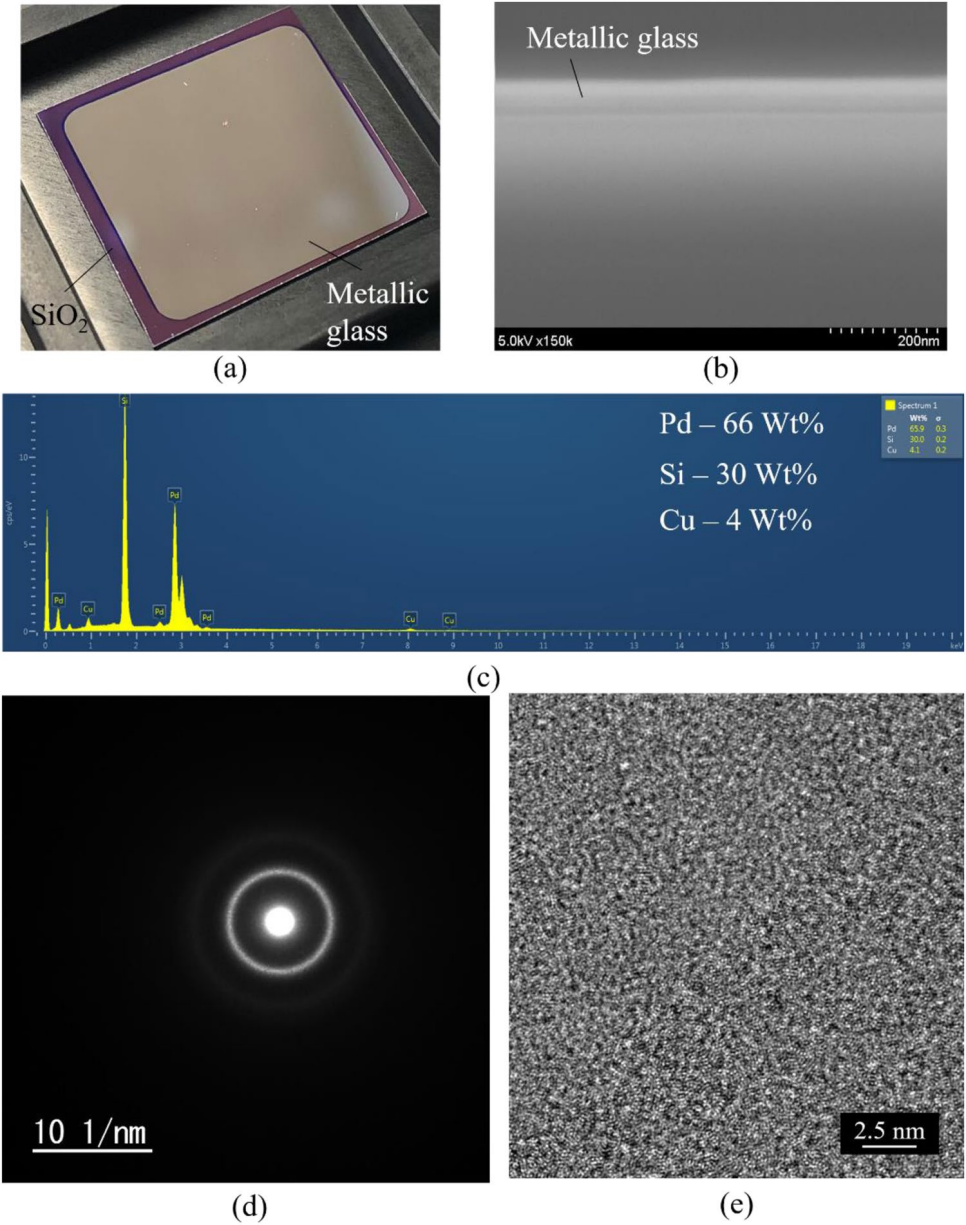
Material analysis. (**a**) Metallic glass thin film deposited on silicon wafer. (**b**) Cross sectional view of metallic glass thin film. (**c**) EDX elemental analysis. (**d**) SAED pattern. (**e**) High-resolution TEM image.

Figure 2 (a,b) show top and cross-sectional view of the sputtered film which shows high uniformity. An ultra-flat and thin (50 nm-thick) metallic glass film has been achieved (Fig. 2 (b)). Energy Dispersive X-ray (EDX) is employed to evaluate the compositions of the deposited film, as given in Fig. 2 (c). The composition ratio in atomic percentage of Pd, Si and Cu is 66%, 30% and 4%, respectively. The deposited film is amorphous structure which evidenced by the presence of diffuse rings in the SAED pattern, as shown in Fig. 2 (d), as well as the lack of any observable atomic ordering in high-resolution TEM image, as presented in Fig. 2 (e).

### Fabrication

**Figure 3:**
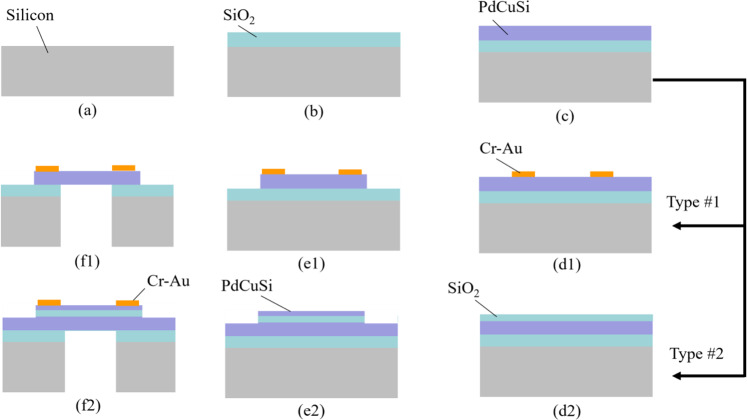
Fabrication process. (**a**) Silicon wafer. (**b**) SiO_2_ deposition. (**c**) Metallic glass sputter. (**d1**) Type #1 – Cr-Au metal electrodes. (**e1**) Type #1 – Metallic glass patterning. (**f1**) Type #1 – deep RIE. (d2) Type #2 – thin SiO_2_ layer deposition. (e2) Type #2 – Metallic glass patterning by lift-off process. (**f2**) Type #2 – deep RIE.

Figure 3 shows the fabrication process for two types of the proposed devices. A 300 µm-thick silicon wafer (Fig. 3 (a)) has been employed as a substrate. A thin SiO_2_ layer with a thickness of 200 nm is deposited on this wafer by CVD as an insulation layer between Silicon substrate and metallic glass thin film to avoid the leakage current to substrate, as well as for the stop layer to etching silicon substrate (Fig. 3 (b)). Next, the 50 nm-thick PdSiCu film is sputtered on the wafer, as shown in Fig. 3 (c).

For type #1: 20-nm-thick Cr and 200 nm-thick Au layers are deposited on entire surface of metallic glass via the sputtering technique. The 3 µm-thick photoresist (OFPR800-200cp, Tokyo Ohka Kogyo Co.) is coated and exposed to ultravisible (UV) light as a conventional photolithography process to make a mask pattern. After the developing process, selective Cr-Au areas are etched by the wet etchant ((Fig. 3 (d1)). The phototoresist is then removed by acetone and rinsed with isopropyl alcohol (IPA). Next, photolithography is employed again to create the mask pattern for etching metallic glass via ion beam milling technology. The metallic glass layer is etched out until reaching to the SiO_2_ layer, as shown in Fig. 3 (e1). Finally, the backside of the wafer is patterned by deep reactive ion etching (RIE) with a combination of sulfur hexafluoride (SF_6_) and octafluorocyclobytane (C_4_F_8_) plasma. The SiO_2_ film is removed by a buffered hydrofluoric (BHF) acid (Fig. 3 (f1)).

For type #2: A thin SiO_2_ layer with a thickness of 50 nm is deposited on metallic glass via the same above-mentioned method (Fig. 3 (d2)). Subsequently, a 10 nm-thick metallic glass film is patterned through the lift-off process (Fig. 3 (e2)). Finally, silicon and SiO_2_ layers are etched out by deep RIE and BHF, respectively, as shown in Fig. 3 (f2).

**Figure 4:**
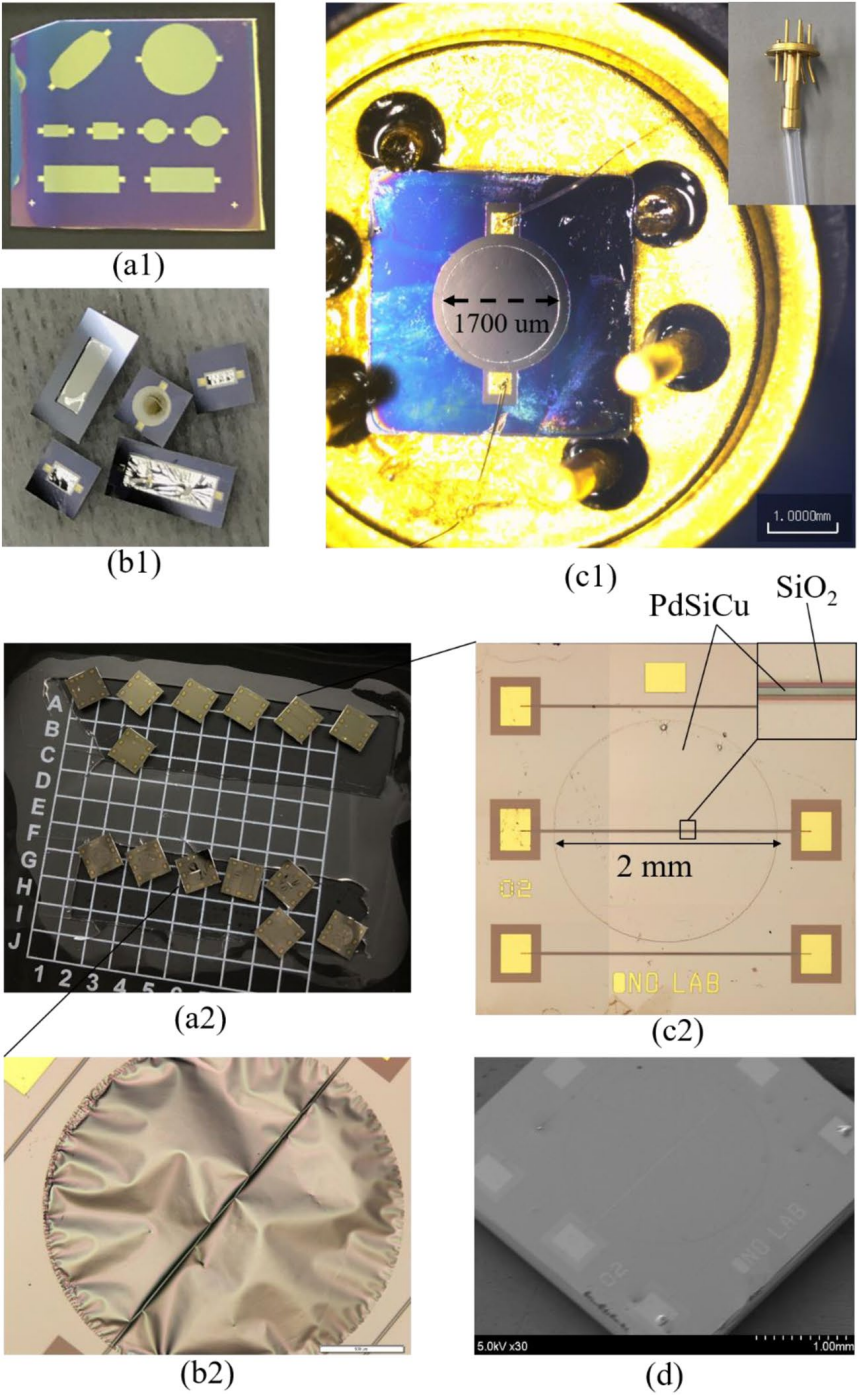
Fabricated results. (**a1**) Type #1 - Metallic glass patterning. (**b1**) Type #1 - Completed device fabrication. (**c1**) Type #1 – mounted on DIP. (a2) Type #2 – Completed device fabrication. (b2) Type #2 – Buckling membrane. (**c2**) Type #2 – Flat membrane. (**d**) Type #2 – SEM image of successfully fabricated device.

**Figure 5:**
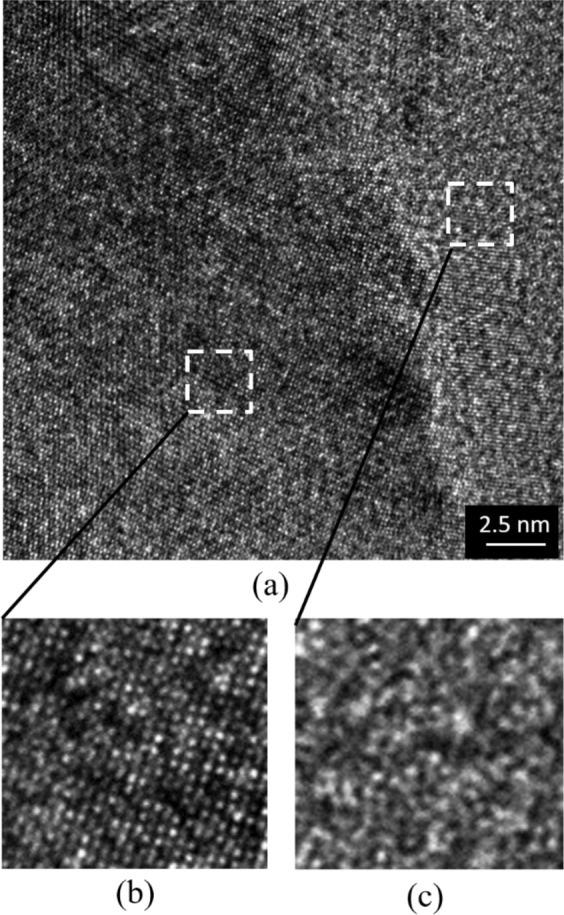
(**a**) HR-TEM for sample after deep RIE process. (**b**) Crystalline structure. (**c**) Amorphous structure.

Figure 4 shows the fabricated results for types #1 (Fig. 4 (a1,b1,c1)) and #2 (Fig. 4 (a2,b2,c2,d)). Several kinds of design have been conducted and fabricated successfully. Patterns are transferred to metallic glass by the ion beam milling etching, as given in Fig. 4 (a1). The fabricated devices of type #1 with freestanding metallic glass membrane as a sensing element are shown in Fig. 4 (b1). Several devices with ultra-flat membrane are produced successfully; however, some devices pose the problem of buckling membrane (Fig. 4 (b1)). One of the main reasons is due to the high temperature process which is created during deep RIE. In this work, in order to perform deep RIE, the 2 cm × 2 cm patterned wafers are bonded on the 4 inch dummy wafer by photoresist. If the bonding between wafer and dummy wafer is not good, there is the possibility of existing air gaps which could generate the high temperature regions on the metallic glass layer. When the temperature is higher than that of the metallic glass transition temperature, amorphous structure is partly changed to crystalline structure. Thus, it could make membrane buckling. The evidence of this claim is shown in Fig. 5 (a). The HR-TEM is investigated for sample after deep RIE process. Amorphous feature is partly change to crystalline feature, as demonstrated in Fig. 5 (b,c). Successful devices are mounted on dual in-lin package (DIP) by araldite and Au wire bonding is performed, as shown in Fig. 4 (c1). The silicone micro-tube is connected to a through hole via of DIP to introduce the pressure from back side of the device.

Figure 4 (a2,b2,c2,d) demonstrate for the experimental results of type #2. 14 devices could be fabricated on 2 cm × 2 cm wafer size. Figure 4 (b2) shows the buckling membrane while Fig. 4 (c2) poses the ultra-flat freestanding metallic glass membrane with the piezoresitive metallic glass bar on the top insulated by the thin SiO_2_ layer. The ultra-large flat and freestanding membranes with 50 nm-thick and 2 mm-diameter have been fabricated successfully (Fig. 4 (d)). The aspect ratio (diameter/thickness) of membrane reaches to 40,000.

### Evaluation

**Figure 6:**
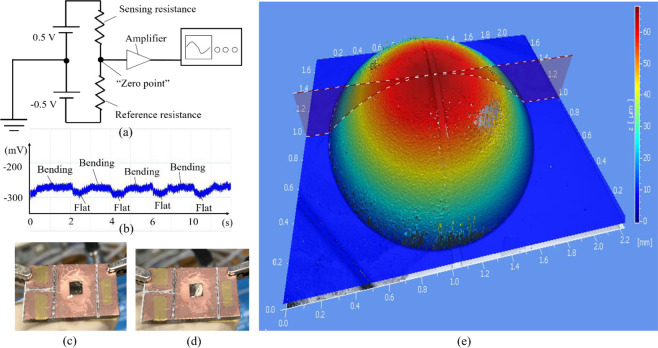
(**a**) Electrical evaluation setup. (**b**) Electrical response. (**c**) and (**d**) Mechanical displacement responding to flat and bending displacement, respectively. (**e**) Mechanical displacement observed by topography measurement system.

The electrical evaluation setup in Fig. 6 (a) is employed to monitor the change of the resistance of the piezoresistive sensing element when the membrane is deformed. Sensing and reference resistances have a similar value because their parameters are the same and they are fabricated at the same time and same conditions. Positive and negative DC sources are employed to create the zero point between sensing and reference resistances. The zero point is then connected to the amplifier with 1000 times. The output signal is monitored by an oscilloscope.

In the case of type #1, no changing output signal is observed. Total resistance of metallic glass membrane (Figs. 1(a) and 4(c1)), including freestanding membrane (resistance #1) and boundary (metallic glass on silicon substrate (resistance #2)) areas, is 91 Ω which is measured by IV curves. Herein, the parallel resistances, consisting of resistances #1 and #2 are considered as equivalent circuit model. The resistance #1 may change its value because of the deforming membrane while the resistance #2 shows no change. However, both resistances have small values, thus make it difficult to observe the change of the electrical response. In addition, the sensing element of type #1 is circular membrane type which means not only the length (increasing resistance) but also the width (decreasing resistance) of the membrane will be possibly expanded when the membrane is deformed. Thus, the changing resistance of sensing element becomes much small. Also, the strain gauge factor of the metallic glass material poses a small value of around 2 which is reported recently in.^36^ Consequently, the output signal of type #1 is hard to the change of the piezoresistive element.

Electrical response is shown in Fig. 6 (b) which indicates the changing resistance of piezoresisitve sensing metallic glass bar without and with applying a pressure on silicone micro-tube which is corresponded to flat (Fig. 6 (c)) and bending (Fig. 6 (d)) membranes. Sensing and reference resistances are the metallic glass bar with parameters of 2300 µm-length, 50 µm-width, and 10 nm-thick. Both elements have the value of approximately 1 kΩ. Although the changing output signal is small, it is still visible observation for flat and bending cases of the membrane. Thus, employing the sensing metallic glass bar on top of the metallic glass membrane, the change of resistance of the piezoresisitve sensing element could be observed. In order to enhance sensitivity, the piezoresisitve sensing bar should reduce into nano scale, such as nano metallic glass beam.

Next, a topography measurement system is employed to observe the displacement of the freestanding metallic glass membrane which is performed at room temperature with a white light interference method. A steady state displacement of the metallic glass membrane is observed by the application of a force on the silicone micro-tube which increases the inside pressure of the micro-tube. The displacement of the membrane can reach approximately 70 µm without damage in the case of the circular membrane diameter of 1700 µm, as shown in Fig. 6 (e). To confirm the maximum displacement until damaged the membrane, a laser displacement system (Keyence, LK-G5000 series) is used. When the displacement of membrane reaches around 110 µm-height, the membrane is broken. Thus, high elastic strain of over 0.7% can be applied without damaged the membrane which is estimated by Eq. 3. The reasons for the high elastic strain and large displacement of the thin metallic glass membrane achieved in this work are possible related to its amorphous atomic structure and a low Young module material. The mechanical characteristic of amorphous material is excellent because of no crystalline defects including grain boundaries and dislocation, compared to those of the crystal structures which results in the high elastic strain. Low Young module can help to achieve the large deflection with a small applied pressure to the membrane. The elastic strain limitation of semiconductor materials is approximately 0.3%, as given in^37,38^ which is 2 times smaller than that of the thin film metallic glass in this work. The Young module of the Pd-based metallic glass thin film is approximately 70 GPa^24^ while that of silicon is 179 GPa.

The sensitivity of the metallic glass membrane and its pressure sensing membrane is calculated by Eq. 1. Herein, in this work, *E* ~ 70 GPa, r = 850 µm and t = 50 nm, the deflection of the circular membrane at the central point is 3.5 µm under the differential pressure between two sides of the membrane of 1 Pa. Thus, the ultra-high sensitivity of the proposed device could be achieved. This examination will be reported in our next works.

Temperature coefficient of resistance *TCR* is an important index for the pressure sensor device. The temperature coefficient of resistance is defined as the ratio between the electric resistance change ratio with the ambient temperature expressed as:

5 $$TCR=\frac{\Delta R/{R}_{0}}{\Delta T}$$

where ∆*R* is the change in material resistance and ∆*T* is the change in ambient temperature.

**Figure 7:**
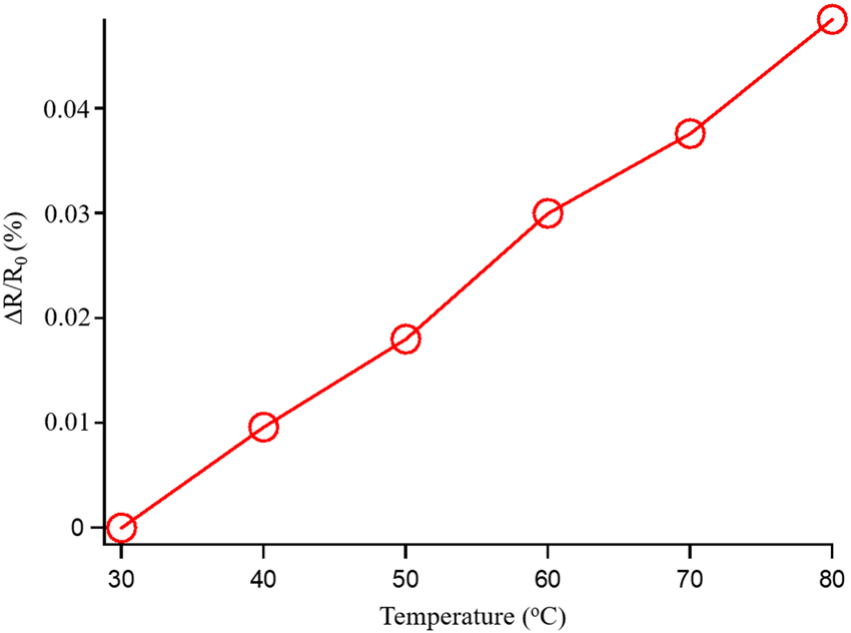
The evaluation result of the resistance change ratio at difference ambient temperature for Pd-based metallic glass piezoresisitve sensing bar.

The measurement is conducted in the temperature controlled chamber with increasing temperature from room temperature to 80 °C. The resistances of the thin film piezoresitive element is monitored by the digital multimeter for estimating the *TCR*. The evaluation result is shown in Fig. 7. According to the Eq. (5), the *TCR* is 9.6 × 10^−6^ °C^−1^. The *TCR* of the Pd-based metallic glass material is much smaller than that of the silicon (7.5 × 10^−2^ °C^−1^ ^37^). The small value of the *TCR* of the metallic glass material would be helpful for the microelectromechanical systems integrated capably with the circuits and the electrical measurement equipment without a thermal drift.

Although thin and flat freestanding metallic glass membranes with the high elastic strain and low *TCR* have been produced successfully, small changing resistance value of the sensing element is an issue which against the fabricated devices toward the real applications. To overcome such this problem, the thin film high strain gauge materials, including Ge_2_Sb_2_Te_5_ (gauge factor of 338,^39^), MoS_2_ (gauge factor of 104,^40^), and V doped MoS_2_ (gauge factor of 140,^41^), should be integrated on thin film metallic glass membranes as highly sensitive elements. Such efforts to prove this are in progress and will be investigated in the next works.

## Conclusion

In this work, the Pd_66_Cu_4_Si_30_ metallic glass thin film has been investigated as the thin metallic glass freestanding membranes toward the highly sensitive micro-fabricated pressure devices. The Pd-based metallic glass thin film is sputtered and evaluated. Its amorphous feature is confirmed by high-resolution TEM and SAED. Two kinds of micro-fabricated pressure sensor devices are designed, fabricated and evaluated. The high elastic strain of over 0.7% can be applied to 50 nm-thick metallic glass freestanding membrane without damaged the membrane. The freestanding membranes could possibly combine with other high strain gauge thin film materials to enhance device performance. This development of ultrathin metallic glass membrane could make micro-fabricated pressure sensor device become a promising candidate for electronic monitoring systems in healthcare, human motion, security, and so on.
